# Cardioprotective Effects of *Aloe vera*-Derived Bioactive Compounds in Myocardial Infarction: A Preclinical Review of Mechanisms and Dosages

**DOI:** 10.3390/life16091397

**Published:** 2026-08-24

**Authors:** Nouf Al-Rawahi, Ali Abduwani, Ayman N. Alhabsi, Abdullah Al Lawati, Hanan Al Lawati, Srijit Das

**Affiliations:** 1 Department of Human & Clinical Anatomy, College of Medicine & Health Sciences, Sultan Qaboos University, Al Khoud, Muscat 123, Oman; noufiyalrawahi@gmail.com (N.A.-R.); s.das@squ.edu.om (S.D.); 2 Department of Medicine, Royal College of Surgeons, D02 YN77 Dublin, Ireland; aymanalhabsi21@rcsi.com; 3 Department of Surgery, Sultan Qaboos University Hospital, Al Khoudh, Muscat 123, Oman; s131482@student.squ.edu.om; 4 Pharmacy Program, Oman College of Health Sciences, Muscat 123, Oman; allawati@ualberta.ca

**Keywords:** *Aloe vera*, antioxidant, cardiovascular, myocardial infarction

## Abstract

Myocardial infarction (MI) is characterized by sudden cardiomyocyte death due to impaired blood supply and remains a leading cause of mortality despite advances in management. *Aloe vera*, a plant rich in over 75 bioactive compounds, including vitamins, minerals, polysaccharides, and anthraquinones, has been widely used in traditional medicine and modern healthcare. Increasing evidence supports its cardioprotective potential through multiple mechanisms. *Aloe vera* and its derivatives have demonstrated antioxidative, anti-apoptotic, anti-inflammatory, antimicrobial, immunomodulatory, and vasodilatory effects relevant to MI pathophysiology. Compounds such as aloe-emodin, emodin, aloin, barbaloin, and selenium-enriched polysaccharides have been shown to modulate pathways including Nrf2/HO-1, TGF-β/SMAD, ERK, ferroptosis inhibition, ionic pump activity, and microRNA regulation. These molecular effects translate into reductions in oxidative damage, apoptotic signaling, inflammatory cytokine release, calcium imbalance, and creatine kinase/LDH leakage, while preserving myocardial structure and function in preclinical models. Collectively, preclinical studies suggest the potential cardioprotective effects of *Aloe vera*-derived preparations and compounds; however, robust clinical evidence in myocardial infarction is lacking, and their therapeutic relevance remains uncertain.

## 1. Introduction

Myocardial infarction (MI) is a vascular pathology that is characterized by sudden death of the cardiomyocytes due to impaired blood supply to the heart tissue. MI is a leading cause of mortality [1]. In the last two decades, the acute MI-related mortality rate has decreased, owing to advances in treatment and management. However, MI remains a significant cause of heart failure (HF), which is associated with devastating rates of morbidity and mortality [2]. Many factors play a role in the pathophysiology of post-MI-related HF, including myocardial damage, stress on the ventricular wall, inflammation, oxidative damage, and neurohormonal factors [3]. Despite advances in the pharmacological management of MI, some agents have limited efficacy and are associated with significant adverse effects with long-term use. Therefore, the scientific community has shifted towards the use of phytomolecules rather than synthetic agents, which have proven to be safe and effective with fewer adverse effects [4]. Several plant-derived compounds have demonstrated cardioprotective potential in experimental MI models. *Aloe vera* contains cardioprotective phytomolecules [5].

*Aloe vera*, also known as *Aloe barbadensis Miller*, is a perennial xerophyte belonging to the Asphodelaceae (formerly Liliaceae). It is typically stemless or has a very short stem. The plant features elongated and pointed leaves that are adapted to store large amounts of water [6].

**Figure 1:**
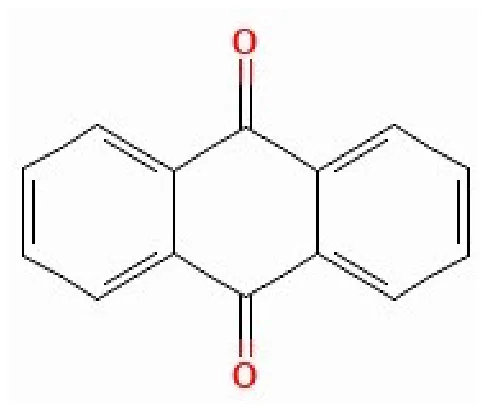
Chemical structure of anthraquinones [8].

Importantly, the plant contains more than 75 compounds, including vitamins, minerals, enzymes, amino acids, fatty acids, sugars, and anthraquinones, making it useful for treating various diseases and disorders [7]. Anthraquinone compounds, such as aloin, aloe-emodin, and emodin, are primarily found in latex or whole-leaf extracts, where they contribute several biological effects, including antioxidant, anti-inflammatory, and cardioprotective actions. However, they have also been associated with dose-dependent toxicity and adverse effects, highlighting the importance of preparation safety evaluation when interpreting their therapeutic potential [6]. Figure 1 illustrates the chemical structure of anthraquinone [8]. Several bioactive constituents of these herbs are known to modulate oxidative stress, inflammation, and apoptotic pathways, which are central to myocardial injury following infarction. Collectively, these properties highlight the role of *Aloe vera* in attenuating myocardial damage, preserving cardiomyocyte viability, and protecting cardiomyocytes from ischemia-induced injury [5].

**Figure 2:**
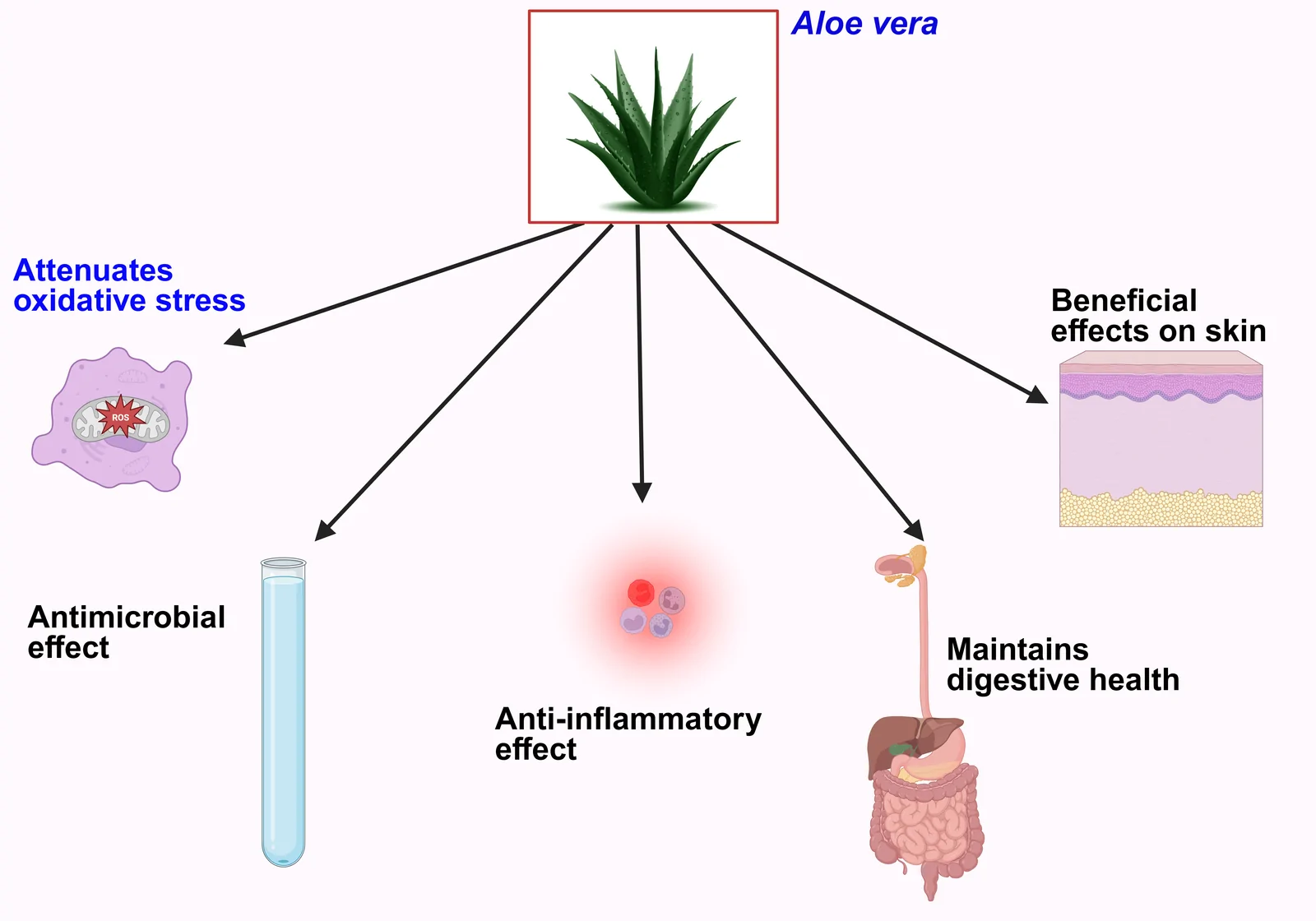
Overview of therapeutic effect of *Aloe vera*. Created with BioRender.com.

A previous mini-review examined the potential protective effects of *Aloe vera* gel against cardiovascular diseases [9]. Recently, broader updates have summarized the phytochemical diversity and systemic therapeutic potential of *Aloe vera* in multiple organ systems [10]. However, pathway-oriented synthesis specifically centered on MI and its underlying molecular mechanisms remains limited in the literature. Therefore, we aimed to synthesize a descriptive analysis of the published clinical evidence on the cardioprotective mechanisms of *Aloe vera* in myocardial infarction, highlighting its effects on key cellular and molecular pathways involved in different medical conditions, while emphasizing their specific relevance to myocardial injury, repair, and remodeling. Figure 2 shows the overall therapeutic effects of *Aloe vera*. However, the available evidence remains predominantly preclinical, and robust clinical evidence supporting the use of *Aloe vera* in myocardial infarction is currently lacking. Therefore, the translational relevance of these findings remains uncertain.

## 2. Search Strategy

A structured narrative search of the literature was conducted using PubMed, Scopus, Google Scholar, and ClinicalTrials.gov to identify studies evaluating the cardioprotective and mechanistic effects of *Aloe vera* and its bioactive constituents in myocardial infarction and related models of myocardial injury. The search covered studies published up to 2025, with emphasis on articles published within the last five years, while older mechanistic studies were included when directly relevant to the review topic. Search terms included “*Aloe vera*,” “Aloe barbadensis,” “Aloe barbadensis Miller,” “myocardial infarction,” “myocardial ischemia,” “ischemia-reperfusion injury,” “cardioprotection,” “cardiotoxicity,” “cardiac remodeling,” “aloe-emodin,” “emodin,” “aloin,” “barbaloin,” “rhein,” “acemannan,” “selenium-enriched *Aloe vera* polysaccharide,” “oxidative stress,” “apoptosis,” “inflammation,” “Nrf2/HO-1,” “TGF-β/SMAD,” “AMPK,” “ferroptosis,” “microRNA,” “CK-MB,” and “LDH.” The principal Boolean search string was as follows: (“*Aloe vera*” OR “Aloe barbadensis” OR “aloe-emodin” OR emodin OR aloin OR barbaloin OR “selenium-enriched *Aloe vera* polysaccharide”) AND (“myocardial infarction” OR “myocardial ischemia” OR “ischemia–reperfusion injury” OR cardioprotection OR cardiotoxicity OR “cardiac remodeling”), with additional mechanistic terms combined as appropriate and syntax adapted for each database. Reference lists of relevant articles were also screened to identify additional studies.

Given the limited direct clinical evidence and the predominantly preclinical nature of the available literature, a structured narrative approach was selected to integrate experimental, mechanistic, translational, and limited clinical findings. Eligible research included English-language peer-reviewed original studies, preclinical in vivo and in vitro studies, clinical trials, observational studies, systematic reviews, and major review articles relevant to *Aloe vera*, its derivatives, and cardiovascular or myocardial injury pathways. Conference abstracts, editorials, letters, non-peer-reviewed sources, and articles without accessible full texts were excluded unless used only for background context. The literature search was last updated on July 20, 2026. As this was a narrative review, formal record counts and a PRISMA flow diagram were not included. Studies were selected based on the title, abstract, and full-text relevance to the stated eligibility criteria.

The available literature is predominantly composed of small preclinical studies reporting beneficial effects, with limited neutral, negative, or contradictory cardiovascular findings. A formal risk of bias assessment was not performed because this was a narrative review; therefore, differences in study design, sample size, controls, preparation standardization, and outcome reporting should be considered when interpreting the findings. The predominance of positive results may also reflect publication bias.

## 3. *Aloe vera* Preparations and Standardization

The biological activity of *Aloe vera* depends on many factors such as the part of the plant being used, extraction method, processing conditions, and final formulation [6,11]. For instance, the inner leaf gel is rich in polysaccharides, particularly acemannan, whereas the latex and outer leaf tissues contain higher concentrations of anthraquinones such as aloin, aloe-emodin, and emodin [6,11]. Moreover, aqueous preparations predominantly extract water-soluble polysaccharides, while organic solvents such as ethanol or acetone may concentrate phenolic and anthraquinone compounds [11]. In addition, processing methods, such as heating, filtration, decolorization, and freeze-drying, can alter acemannan content, molecular weight, and the degree of acetylation, thereby affecting its biological activity [12]. Thus, the plant part, extraction method, and processing conditions can all alter the biological activity of *Aloe vera*, meaning that different preparations, including aqueous and organic solvent extracts, freeze-dried gel, isolated compounds, polysaccharide fractions, and commercial capsules, should not be considered directly interchangeable, because their phytochemical composition, efficacy, and safety may differ [6,11]. Therefore, to enhance standardization, future studies should specify the plant part, extraction procedure, solvent, phytochemical profile, acemannan content, and residual anthraquinone concentration [6].

Given this variability, the cardioprotective evidence presented in this review is interpreted according to the individual bioactive compounds rather than *Aloe vera* as a single entity. The principal compounds with preclinical cardiac relevance include aloe-emodin, emodin, aloin, barbaloin, and selenium-enriched *Aloe vera* polysaccharide (Se-AVP), each tested in distinct experimental models, species, routes of administration, doses, and treatment durations.

## 4. Role of *Aloe vera* in Different Conditions

### 4.1. Action Against Oxidative Stress

*Aloe vera* has demonstrated promising therapeutic effects in various conditions. Oxidative stress is a major contributor to cardiac injury because it promotes cardiac fibroblast activation, collagen deposition, and myocardial fibrosis [13]. Aloin, an anthraquinone compound derived from *Aloe vera*, was evaluated in mice with carbon tetrachloride (CCl_4_)-induced liver fibrosis. Aloin reduces oxidative damage, as demonstrated by decreased malondialdehyde (MDA) levels and increased superoxide dismutase (SOD) activity [14]. However, this study used a non-cardiac model and therefore provided only indirect evidence regarding the antioxidant relevance of aloin to MI. Similarly, oral administration of whole *Aloe vera* gel at 400 mg/kg reduced MDA and increased glutathione peroxidase, SOD, and catalase levels in rats with cisplatin-induced hippocampal injury [15]. Although these findings support antioxidant activity, this neurological toxicity model does not directly reproduce myocardial ischemia or infarction. Isolated aloe-emodin also increased glutathione peroxidase and SOD levels in a murine sepsis model [16]. However, systemic inflammation caused by sepsis differs from myocardial injury caused by coronary occlusion or ischemia–reperfusion injury.

More direct cardiac evidence was reported in a rat model of isoprenaline-induced cardiac damage, in which supplementation with whole *Aloe vera* gel increased SOD and catalase activities, reduced nitric oxide and MDA levels, restored glutathione, lowered CK-MB, and improved histological findings [17]. Nevertheless, isoprenaline-induced injury does not fully reproduce human coronary occlusion, and the effects of the complete gel preparation cannot be attributed to its individual constituents. In a rat myocardial ischemia–reperfusion model, the complete Se-AVP preparation increased antioxidant enzyme activity and reduced MDA, CK, and LDH levels [5]. However, the absence of selenium-only and non-selenized polysaccharide control groups prevents these effects from being attributed specifically to selenium. Collectively, these studies demonstrate model-specific antioxidant effects, with the most direct cardiac evidence arising from the isoprenaline and ischemia–reperfusion models. Their clinical relevance remains uncertain because pharmacokinetic data, myocardial exposure measurements, and evidence from patients with MI are lacking in the literature.

### 4.2. Anti-Apoptotic

*Aloe vera* has promising therapeutic potential owing to its anti-apoptotic effects, particularly in the context of MI. This property is largely attributed to its bioactive compounds, such as aloe-emodin (AE) and emodin, which have been shown to play a critical role in reducing apoptosis. In an in vitro and in vivo study investigating the cardioprotective effects of AE, researchers found that AE significantly mitigated apoptosis in cardiac cells by modulating the balance between pro-apoptotic Bax and anti-apoptotic Bcl-2 proteins, preserving mitochondrial membrane potential, and upregulating microRNA-133, a molecule with cardioprotective and anti-apoptotic properties [18]. The upregulation inhibited caspase-3 activation, a key executor enzyme in the apoptotic pathway. Consequently, in MI-induced mouse models, AE treatment improved cardiac function and decreased infarct size [18]. A recent study that examined the impact of AE on myocardial tissue after MI provides further evidence supporting this. In this study, AE significantly decreased cardiomyocyte apoptosis by downregulating caspase-3 activity, reducing oxidative stress, and inhibiting markers of cardiac hypertrophy, such as atrial natriuretic peptide (ANP), brain natriuretic peptide (BNP), and β-myosin heavy chain (β-MHC) levels. AE also preserves mitochondrial integrity by suppressing the elevation of the Bax/Bcl-2 ratio, thereby reducing mitochondrial-mediated apoptosis and preventing cardiac cell death [19]. In addition to aloe-emodin, emodin, another anthraquinone compound structurally related to AE, has also shown cardioprotective effects in a murine MI model. Emodin exerts anti-apoptotic activity by inhibiting caspase-3 activation and suppressing inflammatory mediators, such as TNF-α and NF-κB, in infarcted myocardial tissue [7,20]. These findings highlight the common mechanism of action of anthraquinone derivatives in inhibiting cardiomyocyte apoptosis and indicate the wider therapeutic potential of the bioactive components of *Aloe vera* in MI treatment. Overall, *Aloe vera* has strong anti-apoptotic properties that protect against myocardial injury and support cardiac function. However, these findings are mainly derived from studies on isolated aloe-emodin or emodin tested in cellular and rodent models, and their achievable plasma or myocardial concentrations following oral *Aloe vera* administration remain unknown. Further pharmacokinetic and clinical studies are required before these anti-apoptotic effects can be considered relevant to human MI.

### 4.3. Antimicrobial (Bacterial, Fungal, Viral)

*Aloe vera* has shown great promise as a natural agent with significant antimicrobial properties, making it a potential alternative to conventional treatments against bacterial, viral, and fungal infections. A cross-sectional study evaluated the antibacterial activity of an *Aloe vera* ethanol-based leaf extract against *Staphylococcus aureus* and *Enterobacterales*. The extract demonstrated clear zones of inhibition 17 mm for *Enterobacterales* and 13 mm for *S. aureus*, indicating notable antibacterial efficacy [21]. Additionally, the study reported minimum inhibitory concentration (MIC) and minimum bactericidal concentration (MBC) values: for *S. aureus*, MIC was 94 ± 41.23 mg/mL, and MBC was 188 ± 82.46 mg/mL; for *Enterobacterales*, MIC was 45.6 ± 20 mg/mL and MBC was 91.18 ± 40 mg/mL [21]. These findings support *Aloe vera* ethanol extract as a viable natural antimicrobial agent. Another study investigated various *Aloe vera* gel extracts, including leaf, root, ethanol-based, and non-ethanol-based, against common pathogens such as *Escherichia coli*, *Shigella*, *Salmonella* spp., and *S. aureus* [22]. Ethanol-based extracts (both leaf and root) were the most effective against Gram-negative and Gram-positive bacteria, while raw (non-ethanol) extracts demonstrated moderate activity, suggesting that ethanol enhances the antibacterial compound concentration [22]. Furthermore, a phytochemical analysis comparing freeze-dried leaf gel extract (LGE) and 95% ethanol-extracted leaf gel (EELG), showed that ELGE contained fewer types of compounds but at concentrations approximately 345 times higher than LGE, making it more suitable for in vivo biological efficacy studies. Acetone-based extracts showed the highest antibacterial activity, with inhibition zones ranging from 12 to 20 mm depending on the strain tested [23]. Another study tested the ability of *Aloe vera* gel to decontaminate gutta-percha (GP) cones, showing zones of inhibition of 24 mm (*E. coli*), 21 mm (*E. faecalis*), and 24 mm (*S. aureus*) [24]. These findings from previous clinical trials support *Aloe vera* as a potent, natural antimicrobial agent with promising applications in dental and clinical practice, especially when considered for synergistic use with conventional antibiotics. These antimicrobial findings were derived mainly from laboratory and localized infection models and do not provide direct evidence of cardioprotection in MI. Their relevance is indirect because infection-related systemic inflammation may worsen myocardial injury and post-MI outcomes [25].

### 4.4. Anti-Inflammatory

**Figure 3:**
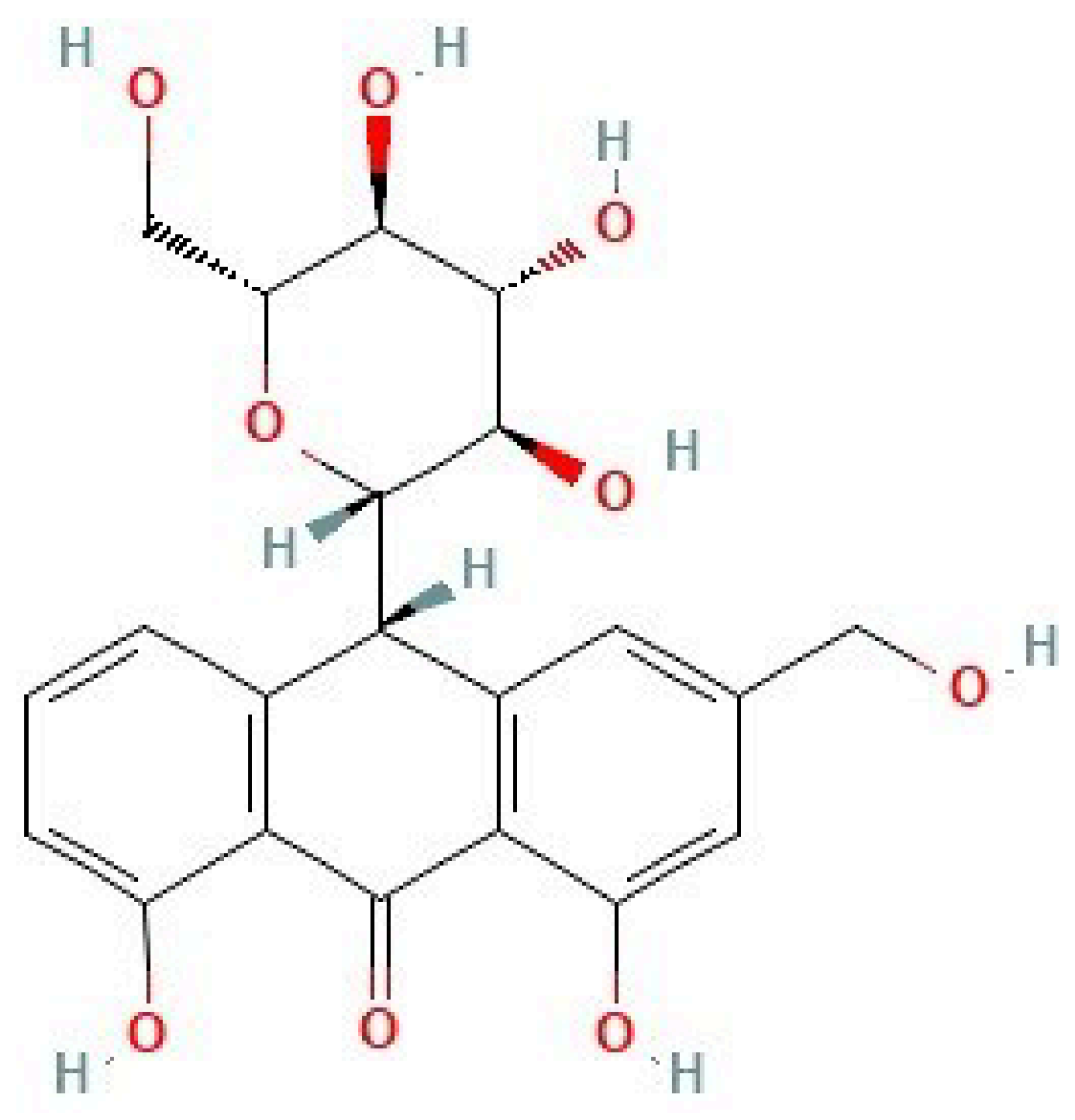
Chemical structure of barbaloin [8].

Notably, *Aloe vera* has demonstrated significant therapeutic potential as a natural anti-inflammatory agent, particularly in cardiac conditions such as MI. A preclinical trial that examined the protective effects of *Aloe vera* gel on the heart in rats with isoprenaline (ISO)-induced myocardial infarction found promising results. The treatment was effective in reducing inflammation by limiting immune cell infiltration, mitigating oxidative stress, and preventing fibrotic remodeling of cardiac tissue [17]. These anti-inflammatory effects are particularly relevant in cases where MI results from acute coronary artery occlusion, which triggers the release of inflammatory cytokines, such as TNF-α, IL-1, and IL-6. These cytokines further aggravate myocardial damage by promoting coronary plaque formation and rupture and are strongly correlated with cardiac dysfunction [26]. In such inflammatory settings, *Aloe vera’s* bioactive compound emodin is very important because it stops TNF-α expression and inhibits NF-κB activation, thereby attenuating local inflammation and minimizing myocardial injury. Furthermore, emodin inhibits the progression of vulnerable atherosclerotic plaques (VAP), and vasodilation mediated through the inhibition of serotonin (5-HT)-induced contraction and enhancement of vasodilation due to acetylcholine, highlighting its cardiovascular protective properties [26]. Similarly, another key compound in *Aloe vera*, barbaloin, exhibits anti-inflammatory effects. In a rat myocardial ischemia/reperfusion (MI/R) model, oral barbaloin pretreatment at 20 mg/kg/day for five days conferred cardioprotection by activating the AMPK signaling pathway, which regulates energy metabolism and inflammation [27]. However, this evidence is preclinical, and no human pharmacokinetic or dose translation data are available. The chemical structure of barbaloin is shown in Figure 3 [8]. Similarly, aloin, another anthraquinone compound from *Aloe vera*, protects against MI/R injury. It exerted potent anti-inflammatory effects by downregulating pro-inflammatory cytokines (TNF-α, IL-6, IL-1β), suggesting that aloin may serve as a promising therapeutic candidate for treating MI and related ischemic conditions [28]. Additional evidence supports the anti-inflammatory properties of *Aloe vera* through its suppression of key inflammatory markers (IL-1, IL-6, IL-8, and TNF-α). It also inhibits multiple inflammatory signaling pathways triggered by lipopolysaccharides (LPS), including NF-κB, p38 MAPK, JNK, and ERK, as well as NLRP3 inflammasome activation leading to a substantial reduction in IL-1 production [29]. Overall, preclinical and preliminary data support cardioprotective and anti-inflammatory effects of *Aloe vera*, which continue to be a primary mechanism contributing to its potential clinical use in cardiovascular disease.

### 4.5. Immunomodulatory

*Aloe vera* also exhibits strong biological plausibility as a cardioprotective agent because of its immunomodulatory properties. One of its key bioactive components, acemannan, a polysaccharide, plays a central role in stimulating macrophages to regulate the production of cytokines such as TNF-α, IL-1, IL-6, and IFN-γ. It also enhances antigen presentation and the overall immune response [30,31,32]. In addition, glycoproteins, such as lectins, found in *Aloe vera* contribute to immune cell activation through various immune signaling cascades. Interestingly, *Aloe vera* gel has demonstrated immune-protective effects even when applied post-damage, such as after ultraviolet (UV) exposure. This suggests its capacity to modulate downstream immune signaling pathways, which could be particularly relevant in post-injury inflammatory conditions such as myocardial infarction (MI) [33,34]. One study investigating the effect of *Aloe vera* on macrophages infected with *Candida albicans* showed a dose-dependent and fraction-specific enhancement in macrophage viability and function, supporting the hypothesis that certain *Aloe vera* constituents actively promote immune competence [35]. This finding implies a potential role for *Aloe vera* in improving immune function during the recovery phase following cardiac injury, where efficient macrophage activity is essential for tissue repair and resolution of inflammation. Furthermore, a recent study identified anthraquinones in *Aloe vera,* including emodin, aloin, and aloe-emodin, as potent immune modulators. These compounds regulate immune responses by suppressing pro-inflammatory cytokines (IL-1β, TNF-α, IFN-γ), promoting immune balance through a reduction in Th1/Th17 cells and enhancement in Th2 responses [36]. They also inhibit different signaling pathways involved in inflammation, namely NF-κB, MAPK, and PI3K/AKT, and modulate NLRP3 inflammasome pathways to strengthen the overall natural immunomodulatory properties of *Aloe vera*. Taken together, these findings provide indirect mechanistic evidence, as they were not demonstrated in myocardial infarction models and did not establish cardioprotective efficacy.

### 4.6. Dermatological Effects

*Aloe vera* has been shown to promote wound healing through fibroblast activity, angiogenesis, collagen remodeling, and modulation of inflammatory mediators in dermatological models [37,38,39,40]. However, these findings cannot be directly extrapolated to myocardial repair because skin and myocardial tissues differ substantially in their cellular compositions and healing responses. Therefore, this evidence provides only indirect mechanistic context, and dedicated post-MI studies are required to determine whether similar effects occur in the cardiac tissue [41]. Few preclinical and clinical trials conducted on the effect of Aloe vera on cardiac health are shown in Table 1 [9,17,18,42,43].

**Table 1:** Representative preclinical, experimental, and human studies evaluating *Aloe vera*-derived preparations in cardiac and related conditions. The human studies provide indirect, hypothesis-generating evidence because they involved chronic heart failure and dialysis populations rather than acute myocardial infarction.

Study Type	Study Country and Continent	Formulation/Agent Name	Subjects	Dose/Duration of Treatment	Results Observed	Adverse Effects	Study Limitations	References
Preclinical study	Dhaka, Bangladesh	Isoprenaline (ISO) *+ A. vera* gel	18 Long-Evans male rats were divided equally into three groups (Control group, ISO only, and ISO + *A. vera* gel)	*A. vera* gel (5% gel mixed with powder chow food) and ISO (50 mg/kg subcutaneous for 14 days, twice a week	The level of antioxidant enzymes like superoxide dismutase in the heart was found to be significantly more in the ISO + *A. vera* gel group compared to ISO group only (*p*< 0.05). The glutathione activity in the heart in the ISO + *A. vera* group was significantly higher compared to the ISO only group (*p*< 0.01).	None reported	Small group size (6 rats per group) Short duration (14 days)	[17]
Preclinical study	Lucknow, India	*A. vera* extract in Kreb’s solution	24 male albino rats divided into four groups (control, 100 mg/L, 200 mg/L, and 300 mg/L)	An aqueous *Aloe vera* extract was prepared by dissolving 10 g of fresh *Aloe barbadensis* gel in 100 mL of distilled water, followed by 15 min vertex and filtration.	*Aloe vera* produced a significant, dose-dependent increase in myocardial contractility, as reflected in the groups taking 200 mg/L and 300 mg/L (10.90 ± 1.80 mm to 14.50 ± 2.28 mm and 11.50 ± 1.20 mm to 16.50 ± 1.38 mm, respectively, without significant changes in heart rate. The enhanced contractility was accompanied by a significant increase in coronary flow at higher doses, suggesting improved myocardial perfusion may contribute to the positive inotropic effect.	None reported	Small group size (6 rats per group) No disease model used	[42]
Preclinical study	Harbin, China	Aleo-emodin dissolved in dimethyl sulfoxide	Healthy adult male Kunming divided into four groups: Aleo-emodin, Aloe-emodin + myocardial infarction, myocardial infarction, and control group.	Aloe-emodin (100 mg/kg/day) for two weeks	The MI size was significantly smaller in the MI + AE group (32.22 ± 1.10%) than in the MI group (44.50 ± 3.07%). Aloe-emodin significantly reduced infarct size, improved cardiac function, and decreased apoptosis and oxidative stress in myocardial infarction mice. In vitro, it protected cardiomyocytes from H_2_O_2_-induced apoptosis by reducing ROS and caspase-3 activity.	None reported	Short post-MI observation window (3-day follow-up only); no long-term survival or chronic remodeling data	[18]
Double-blind randomized placebo-controlled clinical trial	Yazd, Iran	*Aloe vera* gel (AVG)	32 patients over 18 years of age with chronic, stable moderate to moderately severe systolic HF were divided into two groups; 16 received AVG, while the other 16 received a placebo.	One 150 mg AVG capsule twice a day for 8 weeks	After 8 weeks, the AVG group showed a marked improvement in quality of life, with the MLHFQ total score decreasing from 60.6 to 29.6, compared with a minimal change in the placebo group (57.8 to 54.4) (*p* < 0.001) NYHA functional class improved in 69.2% of AVG patients vs. 7.1% in placebo (*p* = 0.004), while the 6 min walk distance increased more with AVG (+83.4 m vs. +45.8 m), but was not statistically significant.	Stomachache was reported in one patient in the AVG group.	Small sample size Key markers not assessed at baseline/endpoint (echocardiographic measures, NTproBNP, oxidative stress markers, liver/renal function, electrolytes)	[9]
Double-blind randomized placebo-controlled clinical trial	Sabzevar, Iran	*Aloe vera* capsules	46 patients who were dialyzed with polysulfone capillary dialyzers 3 times a week for 4 h per session divided into two groups; 23 received the *Aloe vera* capsules while the other 23 received placebo.	Two *Aloe vera* capsules (500 mg/day) daily for 8 weeks	Compared with placebo (*n* = 23), *Aloe vera* (*n* = 23) reduced hs-CRP from 29.95 ± 3.96 to 18.86 ± 2.51 mg/L (adjusted −10.72 mg/L; *p* = 0.01), total cholesterol from 141.35 ± 5.83 to 128.16 ± 5.83 mg/dL (*p* = 0.01), and LDL-C from 112.90 ± 4.96 to 102.54 ± 4.74 mg/dL (*p* = 0.02), while increasing HDL-C from 38.32 ± 2.30 to 44.45 ± 2.44 mg/dL (*p* = 0.002); no significant changes occurred in placebo.	None reported	Lack of assessment of the effect of *Aloe vera* supplementation on vascular inflammation markers and apo-lipoproteins	[43]

ISO: Isoprenaline; AVG: *Aloe vera* gel; Hs-CRP: high-sensitivity C-reactive protein.

## 5. *Aloe vera* and Myocardial Infarction

### 5.1. Na^+^/K^+^-ATPase and Ca^2+^/Mg^2+^-ATPase Activities (ATPase Activity and Calcium Dysregulation)

Myocardial ischemia–reperfusion injury (MIRI) occurs when the rapid restoration of blood flow in an ischemic territory during MI causes a paradoxical exacerbation of myocardial damage [44]. During the ischemic phase, cardiomyocytes rely on anaerobic ATP production, which compromises active Ca^2+^ reuptake into the sarcoplasmic reticulum and ATPase-dependent Ca^2+^ efflux across the plasma membrane. Consequently, this contributes to the ionic disruption detected in the MI [45]. In congruence, Hua Li et al. (2018) demonstrated that pNaKtide, a peptide derived from the Na^+^/K^+^-ATPase α1 subunit, attenuates the Na^+^/K^+^-ATPase/Src/ROS amplification loop and hence confers cardioprotection against MIRI, offering a framework for therapeutic strategies for MI [46]. Taken together, these findings indicate that MIRI is associated with a reduction in Na^+^/K^+^-ATPase and calcium–magnesium adenosine triphosphatase (Ca^2+^/Mg^2+^-ATPase) activities. Se-AVP, an *Aloe vera*-derived polysaccharide conjugated with selenium, was evaluated orally at 100, 200, and 400 mg/kg in rats subjected to myocardial ischemia–reperfusion. Treatment was associated with higher myocardial Na^+^/K^+^-ATPase and Ca^2+^/Mg^2+^-ATPase activities, smaller infarct area, improved antioxidant indices, lower CK and LDH levels, and fewer TUNEL-positive cells [5]. These findings suggest that Se-AVP may help preserve ionic homeostasis in this experimental model. However, the study did not include selenium-only, selenium-free *Aloe vera* polysaccharide, or non-selenized polysaccharide comparison groups; therefore, the observed effects cannot be attributed specifically to selenium or interpreted as evidence that ionic pump modulation is a central mechanism. Pharmacokinetics and tissue distribution were also not assessed, and it remains uncertain whether the intact Se-AVP macromolecule or selenium reaches the myocardium after oral administration. Comparative studies using non-selenized polysaccharides and measurements of plasma and myocardial exposure are required before the clinical relevance of these findings can be determined. Beyond Se-AVP’s preservation of ion transport enzyme activity, AE demonstrated efficacy in alleviating myocardial calcium imbalance induced by a high-fat diet through regulation of the protein arginine methyltransferase 1 (PRMT1)/calcium/calmodulin-dependent protein kinase II (CaMKII) signaling pathway. In high-fat diet-induced cardiomyopathy, a condition that exacerbates myocardial susceptibility to ischemic injury, suppression of PRMT1 and activation of CaMKII impair calcium homeostasis by reducing calcium transients, prolonging calcium decay, and elevating resting intracellular calcium levels. AE reversed these alterations by restoring PRMT1 expression and normalizing CaMKII signaling. Additionally, AE reestablished the balance of calcium-handling proteins by decreasing the overexpression of ryanodine receptor-2 (RyR2) and sodium-calcium exchanger (NCX), while increasing the expression of sarco/endoplasmic reticulum Ca^2+^-ATPase 2a (SERCA2a). Overall, these molecular modifications prevent calcium overload and preserve cardiomyocyte excitation–contraction coupling [47], underscoring a mechanistically distinct yet complementary pathway through which *Aloe vera*-derived compounds safeguard myocardial integrity in MI.

### 5.2. Regulation of microRNA Activity

Extending these cardioprotective mechanisms, AE has been shown in both in vivo and in vitro models of MI to prevent cardiomyocyte apoptosis through the upregulation of microRNA-133 (miR-133) and inhibition of oxidative stress. In neonatal rat ventricular myocytes (NRVMs) exposed to hydrogen peroxide (H_2_O_2_), AE reduced apoptosis by restoring the balance of B-cell lymphoma 2 (Bcl-2) and BCL2-associated X protein (Bax), preserving mitochondrial membrane potential, suppressing reactive oxygen species (ROS) accumulation, and attenuating caspase-3 activation. Similar findings were observed in murine models of coronary artery ligation. AE treatment reduced infarct size, improved ejection fraction and fractional shortening, alleviated myocardial fibrosis and inflammation, and lowered serum levels of lactate dehydrogenase (LDH) and creatine kinase (CK). Mechanistically, inhibition of miR-133 abolished AE’s antioxidant and anti-apoptotic actions, implicating miR-133 as a pivotal mediator, with caspase-3 identified as a downstream effector. Collectively, these findings support a model in which AE protects against MI through miR-133-dependent regulation of antioxidant defense, mitochondrial stability, and apoptosis. However, the precise upstream mechanism by which AE upregulates miR-133 expression remains unclear [18]. These findings support model-specific regulation of miR-133 by isolated AE. However, the evidence remains preclinical, and pharmacokinetic studies are required to determine whether effective myocardial exposure can be achieved in humans. Similarly, emodin has been shown to protect against MIRI through the regulation of miR-217. In rat models of MIRI, the long non-coding RNA X-inactive specific transcript (XIST) was upregulated, leading to suppression of miR-217 and consequent elevation of histone deacetylase 4 (HDAC4). This cascade promotes oxidative stress, apoptosis, and myocardial injury. Emodin treatment downregulated XIST, thereby restoring miR-217 levels and suppressing HDAC4 expression. This resulted in reduced infarct size, lower cardiac enzyme release, decreased oxidative stress markers, reactive oxygen species (ROS), and malondialdehyde (MDA), and downregulation of pro-apoptotic proteins (Bax and caspase-3). In addition, emodin enhanced the antioxidant defenses of SOD and glutathione peroxidase (GPx), and upregulated Bcl-2 expression. Notably, overexpression of XIST abolished these protective effects, confirming that emodin confers cardioprotection through the XIST/miR-217 axis, with miR-217/HDAC4 identified as a downstream regulatory relationship [48].

In addition to miR-133 and miR-217, emodin regulates miR-138, thereby protecting cardiomyocytes from hypoxia-induced injury. In H9c2 cells, a rat embryonic cardiomyoblast line, hypoxia decreased cell viability, increased apoptosis, and upregulated pro-apoptotic proteins, including cleaved caspase-3 and caspase-9. Emodin treatment substantially ameliorated these effects by improving viability, reducing apoptosis, suppressing hypoxia-induced increases in p53 and p21, and restoring cyclin D1 expression. Specifically, emodin upregulated miR-138, which was suppressed by hypoxia. Upregulation of miR-138 alleviated hypoxia-induced cell injury and enhanced the protective effects of emodin, whereas inhibition of miR-138 negated these effects. Further analysis demonstrated that miR-138 directly targets mixed lineage kinase 3 (MLK3), thus activating the Sirt1/AKT and Wnt/β-catenin signaling pathways. These findings reveal that emodin mitigates hypoxia-injured cardiomyocytes through miR-138-mediated inhibition of MLK3 and subsequent activation of survival signaling pathways [49]. In addition to direct myocardial protection, emodin modulates vascular remodeling through microRNA regulation. In a rat model of balloon-injured carotid artery, Hua et al. reported that emodin markedly attenuated intimal hyperplasia by reducing arterial wall thickening, vascular smooth muscle cell (VSMC) proliferation, and collagen deposition. Mechanistically, emodin suppressed the Wnt4/Dvl-1/β-catenin signaling cascade, which is known to drive restenosis, while simultaneously upregulating the endothelial-specific microRNA, miR-126. In vitro, emodin reversed angiotensin II (Ang II)-induced VSMC proliferation and Wnt4/Dvl-1/β-catenin pathway activation by elevating miR-126 levels. These effects were replicated using miR-126 mimics and abrogated by miR-126 inhibitors. This confirmed the central regulatory role of miR-126 in mediating emodin’s inhibition of collagen expression and VSMC proliferation [50]. Although this study primarily focused on vascular injury and restenosis, suppression of Wnt/β-catenin signaling and upregulation of miR-126 by emodin may also have implications for MI, where abnormal VSMC proliferation, fibrosis, and maladaptive remodeling contribute to post-infarct pathology. Despite these promising effects, the therapeutic use of miRNAs remains limited by delivery challenges, such as poor stability, and rapid degradation in vivo, underscoring the need for advanced delivery platforms like lipid nanoparticles and scaffold-based systems [51].

### 5.3. Creatine Kinase (CK), CK-MB, and LDH Leakage

In an in vivo rat model of isoprenaline (ISO)-induced MI, *Aloe vera* gel supplementation exhibited marked cardioprotective effects. ISO administration increased oxidative stress by elevating MDA, nitric oxide (NO), and advanced protein oxidation products (APOPs), and reducing the antioxidant defenses of SOD, catalase (CAT), and glutathione (GSH), while also elevating creatine kinase-MB (CK-MB) and myeloperoxidase (MPO) activities. *Aloe vera* reversed these changes by restoring antioxidant enzyme activity, replenishing GSH levels in the plasma and heart tissue, and lowering MDA, NO, CK-MB, and MPO levels. Histological analysis confirmed reduced inflammatory infiltration, cardiomyocyte hypertrophy, and ventricular fibrosis. Phytochemical analysis further identified polyphenols, including catechin, caffeic acid, ferulic acid, ellagic acid, and quercetin, suggesting that these compounds underlie the antioxidant and antifibrotic effects of *Aloe vera* [17]. Collectively, these findings demonstrate that *Aloe vera* protects against MI by enhancing antioxidant defense, suppressing inflammation, and attenuating fibrosis [17]. The study did not determine their plasma concentrations, myocardial concentrations, metabolic products, or intracellular exposure. It also did not compare the complete preparation with a preparation lacking these compounds, administer the compounds individually, or use an intervention capable of establishing their causal contribution.

In agreement with this, aloin lowered CK-MB and lactate dehydrogenase (LDH) levels that were markedly elevated by ISO administration, indicating reduced myocardial damage and enzyme leakage [52]. Furthermore, aloin treatment significantly and dose-dependently reduced CK-MB and LDH elevations, and at doses above 5 mg/kg, CK-MB and LDH values approached those of control animals. Similar trends were observed for transaminases (AST and ALT), where aloin produced significant reductions across all tested doses, beginning with a low dose of 1 mg/kg. These findings indicate that aloin attenuates the biochemical markers of myocardial injury in a doxorubicin-induced cardiotoxicity model, as reflected by the normalization of key biochemical markers of cardiac injury [53]. While valuable, it is crucial to distinguish chemotherapy-induced cardiotoxicity from ischemic MI, as the underlying pathophysiology and treatment implications differ. Likewise, an in vitro study investigated the cardioprotective role of aloin in H9c2 cardiomyocytes, a rat heart-derived cell line commonly used to model cardiac injury, exposed to simulated I/R injury. Aloin preserved cell viability and reduced apoptosis by suppressing Bax expression while enhancing Bcl-2 expression. It attenuates oxidative stress by lowering ROS, LDH, and MDA, and by restoring SOD activity [28]. Consistent with these results, treatment with emodin significantly reduced CK, LDH, and HBDH levels, indicating protection against cardiomyocyte damage [48].

Se-AVP supplementation significantly prevented the increase in CK and LDH. By limiting the leakage of these myocardial markers, Se-AVP effectively protected against cardiomyocyte damage and further supporting its role in reducing MIRI in MI. This protective effect reflects the preservation of cardiomyocyte membrane integrity, thereby limiting structural damage and the leakage of cardiac enzymes into the circulation [5]. Pretreatment with Barbaloin (Bar), the primary active ingredient in *Aloe vera*, one week before ischemia, significantly reduced myocardial injury, as indicated by lower serum levels of LDH and CK, decreased infarct size, and reduced cardiomyocyte apoptosis. Mechanistically, Bar inhibited endoplasmic reticulum stress (ERS) by suppressing the upregulation of GRP78 and caspase-12 and downregulating canopy homolog 2 (CNPY2), which was shown to initiate the PKR endoplasmic reticulum kinase (PERK) and C/EBP-homologous protein (CHOP) apoptotic signaling pathways. Bar treatment markedly reduced the expression of PERK, phosphorylated PERK, ATF4, CHOP, and cleaved caspase-3, thereby attenuating ERS-mediated apoptosis. Bar protects against MIRI in MI by inhibiting the CNPY2-PERK-CHOP pathway, highlighting ERS suppression as its main mechanism of cardioprotection [54]. Additionally, while CK and LDH are common indicators of cardiomyocyte damage, they are non-specific, and normalization of these markers alone does not fully capture the therapeutic efficacy in MI.

### 5.4. Nrf2-HO-1 Pathway

Extending the mechanistic framework, the regulation of redox-sensitive transcription factors such as nuclear factor erythroid 2-related factor 2 (Nrf2) provides additional insight into the molecular basis of *Aloe vera’s* therapeutic potential in MI. Elevated Nrf2 expression in MI signifies its role as a compensatory response to ischemic injury and affirms its clinical relevance [55]. In this regard, an in vitro model of MIRI using H9c2 cardiomyocytes revealed that aloin exerts its therapeutic effects through activation of the Nrf2/heme oxygenase-1 (HO-1) pathway. Aloin enhanced Nrf2 levels and HO-1 expression, counteracting MIRI-induced oxidative stress and inflammation. This activation reduced ROS accumulation, LDH and MDA release, restored SOD activity, and inhibited pro-inflammatory cytokines TNF-α, IL-6, and IL-1β. It also shifted the balance of apoptotic regulators by lowering Bax levels and increasing Bcl-2 levels, thereby reducing apoptosis. Silencing of Nrf2 attenuated these protective effects, confirming that aloin mitigates MIRI-induced cardiomyocyte injury predominantly via Nrf2/HO-1 activation [28]. Consistently, aloin upregulated Nrf2 and HO-1 expression in myocardial tissue, demonstrating the activation of endogenous antioxidant pathways to counter ISO-induced oxidative stress [52].

Also, AE demonstrated a protective role against doxorubicin (DOX)-induced cardiotoxicity in H9c2 cardiomyocytes through engagement of the Nrf2 signaling pathway. AE treatment upregulated Nrf2 protein expression, enhanced its nuclear translocation, and promoted transcriptional activation in a dose-dependent manner, effects comparable to the canonical Nrf2 activator tert-butylhydroquinone. Activation of Nrf2 subsequently restored the expression of downstream antioxidant genes, including glutathione peroxidase 4 (GPX4) and solute carrier family 7 member 11 (SLC7A11), which had been suppressed by DOX. The restoration of these cytoprotective mediators reduced lipid peroxidation and reestablished redox balance. Importantly, Nrf2 knockdown (si-Nrf2) abolished these protective effects, confirming the necessity of Nrf2 in mediating ferroptosis inhibition. Collectively, these findings indicate that AE alleviates DOX-induced oxidative stress and ferroptosis by restoring Nrf2 activity and its downstream antioxidant defense network [56]. Nonetheless, persistent Nrf2 activation may have unintended consequences, and further studies are needed to evaluate the long-term safety and clinical relevance of these antioxidant mechanisms.

### 5.5. Suppression of the Apoptotic Signaling Pathway

In an integrated in vivo and in vitro study using mouse cardiac injury models and primary cardiomyocytes, AE was shown to protect against MI-induced cardiac remodeling primarily through modulation of the transforming growth factor-β (TGF-β)/SMAD signaling pathway. Specifically, AE suppressed TGF-β1-induced phosphorylation of SMAD2/3 and inhibited non-canonical MAPK signaling (p38 and JNK), while upregulating SMAD7, an inhibitory SMAD that blocks TGF-β receptor-mediated activation of pro-fibrotic signaling. Silencing SMAD7 abolished AE’s antifibrotic effects, confirming that AE exerts its cardioprotective action by preventing TGF-β/SMAD activation via SMAD7 upregulation. Together, these findings establish AE as a regulator of pathological remodeling after MI, acting through the restoration of SMAD7-mediated inhibition of TGF-β signaling [19]. The ability of AE to regulate TGF-β/SMAD signaling represents an emerging mechanism of antifibrotic action in the context of MI. Unlike the general antioxidant or anti-inflammatory effects, this pathway-specific modulation highlights a more targeted role for *Aloe vera-*derived compounds in attenuating post-infarct cardiac remodeling. Given the central role of TGF-β signaling in fibrosis and chronic heart failure progression, AE’s selective interference with this cascade may offer translational value beyond that of conventional cardioprotective agents. In parallel, aloe-emodin may mitigate myocardial infarction-induced apoptosis by reducing oxidative stress and suppressing pro-apoptotic signaling pathways. Consistent with this, aloin pretreatment significantly attenuated SI/R-induced apoptosis in H9c2 rat cardiomyocytes, associated with decreased pro-apoptotic Bax and increased anti-apoptotic Bcl-2 expression, indicating a protective mechanism via modulation of the Bax/Bcl-2 balance [28].

In addition, emodin reduced myocardial apoptosis in a rat post-MI HF model after oral administration at 20, 40, and 60 mg/kg/day for 14 days, mainly by modulating the extracellular signal-regulated kinase (ERK) pathway and preserving mitochondrial function. In post-MI rats, emodin treatment decreased phosphorylated ERK (p-ERK) levels and mitochondrial complex I expression, which are typically elevated with p-ERK, linked to pro-apoptotic signaling. At the same time, emodin lowered serum cardiac troponin I (cTnI), a marker of myocardial injury, and peroxisome proliferator-activated receptor-γ coactivator-1 (PGC-1), reflecting improved regulation of mitochondrial energy metabolism. These combined effects limit cardiomyocyte apoptosis, preserve contractile function, and improve cardiac function in a dose-dependent manner [57]. In parallel, another experimental study demonstrated that rhein reduces myocardial apoptosis primarily through modulation of the mitochondrial fission-mitophagy axis. Rhein inhibits dynamin-related protein 1 (Drp1)-mediated mitochondrial fission, which is typically upregulated after ischemic injury and promotes apoptotic signaling. Simultaneously, rhein enhanced mitochondrial autophagy by upregulating PTEN-induced putative kinase 1 (Pink1), Parkin, Beclin1, and LC3-II/LC3-I, thereby promoting the removal of damaged mitochondria and maintaining mitochondrial integrity. These effects suppressed apoptosis by decreasing the level of pro-apoptotic proteins Bax and cleaved caspase-3 while restoring the level of anti-apoptotic protein Bcl-2. Together, these findings indicate that rhein exerts cardioprotective effects in MI by inhibiting excessive mitochondrial division and activating Pink1/Parkin-mediated mitophagy, thereby preserving mitochondrial homeostasis and preventing cardiomyocyte apoptosis [58].

Furthermore, another pathway investigated by Zhao et al. demonstrated that aloe-emodin protects against zidovudine (AZT)-induced cardiotoxicity by suppressing apoptosis via caspase-dependent signaling. In NRVMs, AZT significantly decreased cell viability, disrupted mitochondrial membrane potential, and increased cleaved caspase-3 expression, all of which were attenuated by co-treatment with aloe-emodin. TUNEL assays further confirmed a reduction in AZT-induced apoptotic cell death with aloe-emodin, indicating its potent anti-apoptotic role. Mechanistically, aloe-emodin activated the p90rsk/p-bad/bcl-2 signaling cascade, leading to the upregulation of the anti-apoptotic protein bcl-2 and downregulation of the pro-apoptotic mediators apaf-1 and cytochrome c. Importantly, inhibition of p90rsk with BI-D1870 abolished these protective effects, highlighting that aloe-emodin exerts its cardioprotective action by suppressing caspase-3 activation via p90rsk-mediated signaling [59]. In addition, Se-AVP protects against ischemia–reperfusion injury by reducing oxidative stress-induced cardiomyocyte apoptosis, as evidenced by the suppression of myocardial apoptotic signaling [5]. Finally, Bar was found to inhibit cardiomyocyte apoptosis by regulating the ER stress-mediated CNPY2-PERK pathway. Bar downregulated the expression of CNPY2 and attenuated the activation of PERK, ATF4, and CHOP, which ultimately led to reduced caspase-3 cleavage. Through modulation of this signaling cascade, Bar effectively suppressed apoptosis and preserved myocardial cell survival during reperfusion injury [54]. However, these findings remain compound- and model-specific, and further pharmacokinetic and clinical studies are required to establish whether comparable effects occur in patients with myocardial infarction.

### 5.6. Action on Pro-Inflammatory Signaling

Bar protects against MIRI in rats by activating adenosine monophosphate-activated protein kinase (AMPK) signaling. Bar upregulates PGC-1α and GLUT4, restores SOD activity, reduces superoxide and MDA accumulation, suppresses pro-inflammatory cytokines TNF-α, MCP-1, IL-6, and restores the anti-inflammatory cytokine IL-10. Inhibition of AMPK with compound C abolishes these antioxidative, anti-inflammatory, and cardioprotective effects, confirming the AMPK-dependent mechanism of Bar [27].

Similarly, aloin demonstrates strong cytokine-modulating actions. SI/R significantly increased the mRNA expression and secretion of pro-inflammatory mediators TNF-α, IL-6, and IL-1β. Aloin treatment markedly suppressed these elevations at both the transcriptional and protein levels, indicating its anti-inflammatory potential through the downregulation of key pro-inflammatory cytokines [28]. Consistently, aloin treatment reduced inflammatory cell infiltration, preserved myocardial architecture, and diminished collagen deposition. Mechanistically, aloin suppresses TGF-β/pSmad2/3 signaling and decreases the fibrotic proteins such as collagen I, α-SMA, and fibronectin, thereby exerting anti-inflammatory and antifibrotic effects [52].

In further support of its anti-inflammatory role, aloin has also been shown to counteract cytokine-driven cardiotoxicity. Aloin exerted a distinct inhibitory effect on doxorubicin-induced inflammatory response. Cardiac tissue from doxorubicin-treated rats showed significant elevations in TNF-α, IL-1β, and IL-6 levels, confirming the cytokine-mediated inflammatory component of cardiotoxicity. Aloin administration significantly reversed these increases, with suppression evident even at the lowest tested dose of 1 mg/kg. Histopathological examination supported these biochemical findings, showing attenuation of myocardial inflammatory changes and tissue injury in the aloin-treated groups. Thus, aloin exerts cardioprotection not only by reducing oxidative stress but also by abrogating the pro-inflammatory cytokine response triggered by doxorubicin [53].

In addition to aloin, emodin has emerged as a key anti-inflammatory compound in *Aloe vera*. Shou et al. demonstrated that emodin protects human aortic endothelial cells against hypoxia–reoxygenation injury by restoring key signaling pathways. Hypoxia–reoxygenation reduced PPAR-γ activity and phosphorylation, eNOS phosphorylation, nitric oxide (NO) production, and HSP90/eNOS molecular coupling, while simultaneously elevating pro-inflammatory cytokines, including TNF-α, IL-6, and IL-8. Emodin treatment reversed these effects in a dose-dependent manner, enhancing PPAR-γ and eNOS phosphorylation, improving NO production, reestablishing HSP90/eNOS interactions, and normalizing cytokine levels. Crucially, the protective actions of emodin were abolished by GW9662, a PPAR-γ antagonist, and L-NAME, an eNOS inhibitor, indicating that its endothelial protective effects are predominantly mediated through the PPAR-γ and eNOS signaling pathways [60]. However, because this was an endothelial hypoxia–reoxygenation model rather than an in vivo MI model, the findings should be interpreted as a mechanistic support rather than direct evidence of efficacy in MI.

Besides its protective properties, emodin modulates macrophage responses and inflammasome activation. In vitro, emodin facilitated the polarization of macrophages from the pro-inflammatory M1 subtype to the anti-inflammatory M2 subtype, as evidenced by decreased CD86 expression and increased ARG1 expression in RAW 264.7 macrophages. This shift was accompanied by elevated levels of anti-inflammatory cytokines (IL-6 and IL-10) and reduced expression of pro-inflammatory mediators (IL-1β, TNF-α). In vivo, rats subjected to MI and treated with emodin, or emodin@F-127 hydrogel showed significantly fewer infiltrating CD68^+^ macrophages and an increased proportion of M2 macrophages in the infarcted myocardium. Emodin@F-127 produced the strongest effect, markedly upregulating IL-10 and TGF-β while downregulating TNF-α [61]. These data support an immunomodulatory role for emodin in MI, but the macrophage findings remain partly in vitro and should be viewed as supportive rather than definitive. Furthermore, emodin protects against myocardial I/R injury by inhibiting gasdermin D (GSDMD)-mediated pyroptosis. Treatment reduced infarct size, preserved myocardial structure, and lowered IL-1β release in vivo, while enhancing cardiomyocyte survival in vitro. These effects were mediated through the suppression of the TLR4/MyD88/NF-κB/NLRP3 inflammasome pathway, highlighting emodin’s anti-inflammatory role in limiting myocardial damage [62]. Although the anti-inflammatory effects of *Aloe vera*-derived compounds are promising, their immunomodulatory effects over long-term use and across different pathological contexts remain poorly understood.

### 5.7. Aloe vera and Vasodilation

*Aloe vera* may support vascular health and regulate blood pressure through the activity of several bioactive compounds. One of these compounds, AE, an anthraquinone found in aloe latex, showed the ability to inhibit key vasoconstrictive agents, such as phenylephrine, endothelin-1, and serotonin, leading to a vasodilatory effect and potential blood pressure reduction [63]. In vitro studies have shown that AE selectively inhibits the proliferation of vascular smooth muscle cells while sparing endothelial cells, thereby preserving vascular functions. Furthermore, AE has been proposed as a coating for drug-eluting stents to prevent plaque buildup and restenosis following vascular procedures [63]. A further study using the rat thoracic aorta revealed that AE reduced vasoconstriction induced by phenylephrine, serotonin, and endothelin-1 through the suppression of calcium sensitization, a key mechanism in vascular smooth muscle contraction [64]. At the molecular level, AE inhibited PKCδ, a protein kinase responsible for maintaining tonic tension, and reduced the phosphorylation of myosin light chain (pMLC) and CPI-17, both of which contribute to vascular tightening. These findings support its role in reducing vascular tone and promoting relaxation [64]. In another study, AE was shown to protect the endothelial barrier in hypertensive models by restoring the expression of tight junction proteins ZO-1 and ZO-2, decreasing HMGB1 release, and inhibiting NLRP3 inflammasome activation through increased NLRP3 ubiquitination [65]. These results highlight the potential of AE to reduce inflammation, protect vascular integrity, and restore function in hypertensive conditions.

Further evidence supports its potent antihypertensive effect, with AE causing 26%, 52%, and 79% reductions in mean arterial pressure at doses of 0.5, 1, and 3 mg/kg, respectively, in rats, demonstrating a strong dose-dependent hypotensive effect [66]. Additionally, a study on *Aloe vera* gel reported increased heart rate, myocardial contractility, and coronary blood flow when administered at 200–300 mg/L in aqueous solution, suggesting that its cardiac stimulatory and coronary vasodilatory effects are likely mediated through β-adrenergic receptor activation and sympathetic stimulation [9]. These bioactive effects are further expanded to influence eicosanoid pathways, including prostaglandins and thromboxanes, which are derived from arachidonic acid and regulate both inflammation and vascular tone. While PGE1 and PGI2 are vasodilatory, TxB2 and PGF2α promote vasoconstriction and platelet aggregation, potentially worsening tissue ischemia in burn injuries [67]. Thus, balancing vasodilation and vasoconstriction is critical in wound zones, as studies have shown that blocking thromboxane or adding prostaglandins improves tissue survival and healing. *Aloe vera* has been shown to inhibit thromboxane B2 (TxB2) as seen with Dermaide Aloe^®^ cream, which led to improved tissue perfusion and increased survival in burn and frostbite models [67,68]. In addition, among Aloe’s active components, emodin and emolin are known to inhibit thromboxane synthetase, while salicylic acid, also present in Aloe, inhibits cyclooxygenase (COX) enzymes, reducing the synthesis of both prostaglandins and thromboxanes, thus further modulating inflammation and vascular reactivity [67]. Overall, these studies suggest vasodilatory and vascular effects of isolated aloe-emodin and different *Aloe vera* preparations in ex vivo, hypertensive, and burn models. However, these findings do not directly establish efficacy in myocardial infarction, and their clinical relevance remains uncertain.

### 5.8. Ferroptosis Inhibition

The cardioprotective effects of *Aloe vera* may be due to the inhibition of ferroptosis, a regulated form of cell death that is dependent on iron and occurs because of the accumulation of poisonous lipid peroxides. Iron is important for proper biological function, but excess iron contributes to cellular damage by promoting ferroptosis [69]. Recent in vitro evidence has identified purified aloe-emodin as a ferroptosis inhibitor in H9c2 rat cardiomyocytes exposed to DOX. Aloe-emodin activated the Nrf2 pathway and upregulated SLC7A11 and GPX4, while also complexing divalent iron (Fe^2+^) and altering the expression of iron-regulatory genes [56]. These findings support an anti-ferroptotic effect within this DOX-induced cell culture model. However, they do not establish efficacy in MI or following oral administration of *Aloe vera*, and the achievable myocardial exposure to native aloe-emodin remains unknown. Furthermore, in a study on Severe Acute Pancreatitis (SAP), the traditional Chinese herbal formulation Da Cheng Qi Decoction (DCQD), which contains aloe-emodin among seven other compounds, was shown to inhibit ferroptosis by restoring GPX4 expression and downregulating NOX2, a key enzyme involved in ROS generation [70]. Importantly, NOX2 knockout alone led to induced elevated GPX4 levels and reduced ROS and HMGB1, while combination therapy with DCQD and NOX2 knockout resulted in a synergistic protective effect. Overall, these results demonstrate that aloe-emodin may mediate its protective effects through the NOX2-GPX4 pathway, providing a possible therapeutic target for inflammation-induced ferroptosis [70]. However, in order to confirm both its therapeutic activity and demonstrate inhibition of ferroptosis by *Aloe vera*, further clinical studies are needed. There is no clinical evidence linking *Aloe vera* to anti-ferroptotic effects, particularly in the context of MI.

### 5.9. Ionic Homeostasis and Perfusion

The excessive rise in intracellular calcium and reduced ATPase activity may contribute to myocardial damage. In a preclinical myocardial ischemia–reperfusion study, Wistar rats received oral Se-AVP at 100, 200, or 400 mg/kg [5]. Se-AVP administration was associated with increased myocardial Na^+^/K^+^-ATPase and Ca^2+^/Mg^2+^-ATPase activities and improved antioxidant and cell survival markers. However, the absence of selenium-only and non-selenized polysaccharide controls, together with the lack of pharmacokinetic and myocardial exposure measurements, prevents the effects from being attributed specifically to selenium and limits their clinical interpretation [5].

## 6. Safety, Bioavailability, Pharmacokinetics, and Translational Challenges

Given the growing body of evidence highlighting the cardioprotective effects of *Aloe vera-*derived compounds in experimental MI, careful consideration of safety and translational feasibility is essential. Regulatory assessments by the European Food Safety Authority have concluded that hydroxyanthracene derivatives, including aloe-emodin and emodin, should be regarded as genotoxic and carcinogenic, and have identified safety concerns, particularly for whole-leaf extracts, precluding the establishment of a safe daily intake [71]. Consistent with this position, anthraquinone-rich preparations have been associated with multi-organ toxicities in experimental models, and clinical case reports have described toxic hepatitis and perioperative bleeding following oral *Aloe vera* consumption [72,73,74]. Collectively, these findings underscore that anthraquinone-containing preparations, particularly whole-leaf extracts, pose safety concerns that may limit their suitability for long-term therapeutic use in humans.

In addition, preparations that are high in anthraquinones, such as latex and whole-leaf extracts, could lead to laxative effects that scale with dose, potentially leading to abdominal pain, diarrhea, dehydration, and electrolyte imbalances [6]. Moreover, drug interactions arising from laxative-induced electrolyte disturbances could also be an important concern, although this has not been directly studied in the context of *Aloe vera* use. In addition to the absence of robust long-term safety studies, these issues pose meaningful barriers to the clinical use of *Aloe vera*.

Importantly, the toxicological liability appears to be formulation-dependent. Regulatory analyses suggest that the adverse effects of *Aloe vera* are largely attributable to the hydroxyanthracene derivative content rather than the purified inner-leaf gel itself [75]. In support of this distinction, a 90-day repeated-dose study of a commercially available *Aloe vera* inner-leaf gel preparation reported no treatment-related adverse effects in rodents [76], and standardized *Aloe vera* soft capsule formulations similarly demonstrated no acute, subacute, or genotoxic toxicity in rodent models at doses exceeding the recommended human intake [77]. These data collectively suggest that appropriately processed, low-anthraquinone formulations may exhibit a comparatively improved safety profile compared to whole-leaf extracts, although cardioprotective data specific to such preparations remain limited. Conversely, many mechanistic studies rely on isolated compounds, such as aloe-emodin, at concentrations that may not be achievable through conventional oral supplementation.

Pharmacokinetic limitations further complicate the clinical translation of the findings. Aloe-emodin exhibits poor intestinal absorption, rapid metabolism, and low systemic bioavailability [78], and available clinical pharmacokinetic observations suggest low systemic exposure following conventional oral intake [79]. Although formulation strategies, such as solid dispersion systems, have improved bioavailability in preclinical models [80], their relevance to cardioprotective therapy remains uncertain.

**Figure 4:**
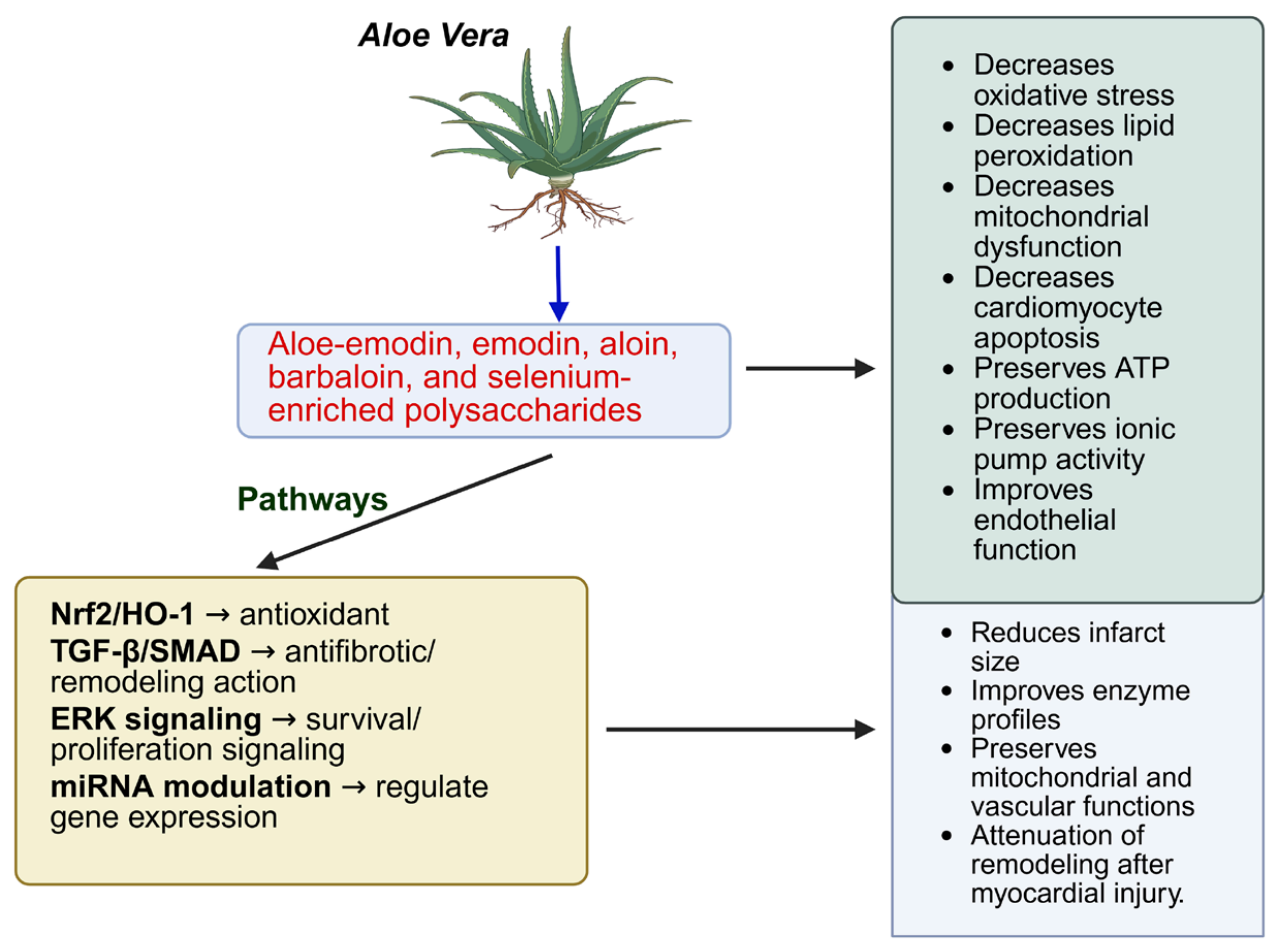
A schematic diagram summarizing the action of different active compounds present in *Aloe vera*. Created with BioRender.com.

Collectively, the cardioprotective effects of *Aloe vera*-derived compounds have primarily been demonstrated in preclinical MI models. Figure 4 summarizes the action of different active compounds present in *Aloe vera*.

Despite promising mechanistic insights, translation to clinical practice is constrained by formulation variability, extensive metabolism, uncertain tissue exposure, anthraquinone-associated toxicity, and the absence of adequately powered clinical trials. Therefore, the current evidence supports hypothesis generation rather than clinical recommendations for MI treatment.

## 7. Conclusions

*Aloe vera* contains diverse bioactive components that act through antioxidant, anti-apoptotic, anti-inflammatory, immunomodulatory, vasodilatory, and ferroptosis-inhibiting mechanisms to protect the myocardium. Preclinical and preliminary clinical studies have demonstrated that aloe-derived compounds such as aloe-emodin, emodin, aloin, barbaloin, and selenium polysaccharides can modulate oxidative stress, apoptosis, ionic pump activity, microRNA expression, and key signaling pathways, including Nrf2/HO-1, TGF-β/SMAD, and ERK. These actions lead to reduced infarct size, improved enzyme profiles, preserved mitochondrial and vascular functions, and attenuation of remodeling after myocardial injury. While conventional therapeutic agents may target a single pathway, the presence of multiple active compounds in *Aloe vera* may prove to have a multitargeted approach, thereby making it a good supplement for treating cardiovascular disorders. While evidence remains predominantly experimental, clinical trials are scarce, pharmacokinetic studies are lacking, and standardized formulations are unavailable, further translational studies are necessary. The pharmacokinetics, bioavailability, long-term safety profile, and potential interactions with other therapeutic agents should be investigated in the future.

## Data Availability

No new data were created or analyzed in this study. Data sharing is not applicable to this article.

## Abbreviations

AE	aloe-emodin
ANP	natriuretic peptide
APOPs	advanced protein oxidation products
Bax	BCL2-associated X protein
Bcl-2	B-cell lymphoma 2
BNP	brain natriuretic peptide
CCl_4_	carbon tetrachloride
CK-MB	creatine kinase-MB
EELG	ethanol-extracted leaf gel
GERD	gastroesophageal reflux
GPx	glutathione peroxidase
GSH	Glutathione
H_2_O_2_	hydrogen peroxide
HDAC4	histone deacetylase 4
HF	heart failure
ISO	isoprenaline
LDH	lactate dehydrogenase
LGE	leaf gel extract
MBC	minimum bactericidal concentration
MDA	malondialdehyde
MI	myocardial infarction
MIC	minimum inhibitory concentration
MMPs	matrix metalloproteinases
NO	nitric oxide
ROS	reactive oxygen species
SOD	superoxide dismutase
VAP	vulnerable atherosclerotic plaques
β-MHC	β-myosin heavy chain
